# Reframing perceptions in operative dentistry relating evidence-based dentistry and clinical decision making: a cross-sectional study among Jordanian dentists

**DOI:** 10.1186/s12903-022-02641-0

**Published:** 2022-12-24

**Authors:** Ayah A. Al-Asmar, Ahmad S. Al-Hiyasat, Nigel B. Pitts

**Affiliations:** 1 Department of Restorative Dentistry, School of Dentistry, University of Jordan, Queen Rania St, 11942 Amman, Jordan; 2 Department of Conservative Dentistry, School of Dentistry, Jordan University of Science and Technology, Irbid, Jordan; 3 Dental Innovation and Impact, Faculty of Dentistry, Oral and Craniofacial Sciences, King’s College, London, UK

**Keywords:** Evidence-based dentistry, Operative dentistry, ICDAS, ICCMS, Esthetic dentistry, Caries

## Abstract

**Background:**

The aim of the current study was to investigate current dental practice in operative dentistry in Jordan, and the relationship between evidence-based dentistry in caries research and decision making in clinical practice in operative dentistry.

**Materials and methods:**

This cross-sectional study was conducted through a survey of dentists in Jordan. The survey aimed to explore the degree of knowledge and practice of evidence-based dentistry in caries research the dentists possess regarding clinical decision making in operative dentistry. The sample size was composed of (5811) dentists whom registered in Jordan Dental Association database. Descriptive statistics were generated and Chi-square test was used to examine associations between the different variables and the significance level was set at P < 0.05.

**Results:**

4000 responses were collected from the web-survey, response rate (68.83%). Nearly half of the surveyed dentists focus on the chief complaint of their patients (n = 2032, 50.8%) rather than doing full mouth assessment. Nearly two-thirds of dentists (n = 2608, 65.2%) treat lesions confined to enamel with operative treatment. Half of dentists use operative treatment when asked about the routine management of radiographically detected proximal caries confined to enamel. When treating incipient lesions, the majority (n = 3220, 80.5%) use preventive treatment. Three-quarters of dentists (n = 2992, 74.8%) treat deep dentinal caries by removing just the soft infected carious dentin, and treated old failed restorations with replacement.

**Conclusion:**

In operative dentistry, the evidence-based research is not implemented clinically. To optimize relationship between evidence-based dentistry and clinical decision-making, dental curriculum has to be updated and modified constantly.

## Background

The practice of dentistry has become increasingly commercialized due to conflicts between the commercial and professional obligations that dental practitioners face every day [1]. The “Daughter Test” in elective esthetic dentistry was suggested by Burke and Kelleher in 2009 [2]. It asks the question “Knowing what I know about what this procedure would involve to the teeth in the long term, would I carry out this procedure on my own daughter?” [3].

From this starting point we can move backward overcoming the overwhelming esthetic fashion and technology in dentistry, toward preserving tooth structure through minimal intervention avoiding the death spiral of the tooth [4]. All restorations have limited life time although they are called “permanent restorations” and once a permanent tooth has been restored, the “restorative cycle” begins through replacing the restoration several times that eventually may lead to destruction of the tooth: the “death spiral” [5]. Accordingly, the main goal of minimal intervention or minimal invasive dentistry (MID) is to increase the life of a tooth, through restoring it conservatively to convey the concept “prevention of extension” rather than “extension for prevention” [6].

Based on the International Caries Detection and Assessment System (ICDAS) which was developed in 2002, assembling a patient-centered personalized health care plan has to be established upon the elements of caries risk assessment together with the classification of caries [7–10]. This paradigm shift in dental care was the foundation of the International Caries Classification and Management System (ICCMS) which was adopted at the Temple University Caries Management Pathways workshop, in 2012 [11]. The ICCMS is a clinical code that provides preservative approaches at the diagnostic, preventive and restorative levels [12]. ICCMS is based on approaches moving towards a preventive / preservative strategy, in which initial caries lesions are prevented and moderate or extensive caries lesions are restored conservatively, rather than the mechanical or restorative care that has been followed around the world [13].

The miscommunication and the big gulf between research findings and clinical practice led to wasting of evidence-based practice in patient dental care. There is a growing need to bridge this gap through formulating evidence-based clinical guidelines for dentists [14]. The American Dental Association defines the term “evidence-based dentistry (EBD),” as an oral health care strategy that requires the sensible integration of systematic assessments of clinically relevant scientific evidence, relating the patient’s oral and medical condition and history, with the dentist’s clinical expertise and the patient’s treatment needs and desires [15].

Unfortunately, clinical practice in restorative dentistry today couldn’t keep pace with the cariology research advancements. The emergence of fundamental concepts in cariology era and restorative field such as prevention, MID, adhesive materials, and esthetic dentistry although came in approximately parallel time lines, the pace of adoption for each was variable. While the research and knowledge focused on prevention and minimum intervention, clinicians and dental markets focused on materials and esthetics. Eventually, practicing dentistry sacrificed prevention and MID for the sake of esthetic demand, commercial dental marketing, and economic status.

The aim of this study was to investigate contemporary dental practice in operative dentistry, and the relationship between EBD in caries research and decision making in clinical practice in operative dentistry.

Null hypothesis: there is no correlation between EBD in caries research and decision making in clinical practice in operative dentistry.

## Materials and methods

The study was conducted as previously described [16] through a structured questionnaire that was generated using SurveyMonkey website to be distributed randomly via web-survey to general practitioners together with specialists in restorative dentistry in different dental sectors. 5406 general dentists and 405 specialists in restorative dentistry are registered in The Jordanian Dental Association (JDA) database. The sample size was composed of (5811) dentists whom we could reach via internet (email, messenger, what’s app). 4000 responses were collected from the web-survey, response rate (68.83%).

The inclusion criteria were: at least two years’ experience as working practitioners, general practitioners together with specialists in restorative dentistry (operative dentistry, endodontics, and fixed prosthodontics).

The questionnaire was validated for validity and reliability through distributing it to 10 dentists (restorative dentistry specialists) out of the sample size. The questionnaire was then modified and adjusted according to these 10 dentists’ feedback. The questionnaire consisted of socio-demographic and professional characteristics such as; year of graduation, name of country/university of bachelor degree graduation, expertise years, and the specialty if one exists.

In terms of EBD as an oral health care strategy that requires the sensible integration of systematic assessments of clinically relevant scientific evidence, relating the patient’s oral and medical condition and history, with the dentist’s clinical expertise and the patient’s treatment needs and desires, the surveyed dentists were asked regarding their management decision regarding the topics mentioned in (Table 1).

**Table 1:** The questions targeted the dentists in the study regarding diagnosis and treatment of patients in Operative Dentistry

Q	Questions’ Statements
1	Do you restrict the treatment to patient’s chief complaint, or perform full mouth charting and patient risk assessment before making decision regarding restoring carious lesion?
2	Do you rely in caries diagnosis on clinical criteria, or clinical criteria with radiographs, or aided other diagnostic tools?
3	Do you treat incipient non-cavitated lesions with preventive non-operative treatment, or with operative treatment?
4	Do you treat discolored occlusal fissures with preventive non-operative treatment, or with operative treatment as class I cavity preparation, or with fissure sealant and/or preventive resin restoration?
5	Do you treat lesions confined to enamel with preventive non-operative treatment, or with operative treatment?
6	Do you routinely restore arrested asymptomatic lesions, or only upon patient’s demand?
7	Do you treat approximal lesion shows on radiograph confined to enamel (localized enamel breakdown without visual signs of dentinal exposure), with preventive non-operative treatment, or with operative treatment?
8	Do you treat deep dentinal carious lesions through removal of soft dentin leaving discolored hard dentin on the floor of deep cavities, or complete removal of soft and hard carious dentin leaving caries-free stain-free floor?
9	Do you routinely replace old failed restorations, or repair them if possible?
10	Do you keep old restorations with no clinical or radiographical signs of failure, or replace them upon patient’s demand?
11	Do you keep and polish old stained restorations (peripheral staining) without clinical symptoms or replace them?

The collected information and responses were coded and statistical analysis was performed using the software SPSS Statistics for Windows, Version 16.0 (SPSS Inc., Chicago, IL, USA). All data were tested for normality using the Shapiro-Wilk test. Descriptive statistics were generated and Chi-square test was used to examine associations between the different variables. The significance level was set at P < 0.05.

## Results

The overall response rate was 68.83% (4000 of 5811 potential participants). The demographic characteristics of the study population are presented in (Table 2).

**Table 2:** Sociodemographic characteristics of the studied sample

Variable	Number (%)
Gender	
Male	1720 (43)
Female	2280 (57)
Country of last degree	
Jordan	2430 (60.8)
Other Arab/Asian countries	830 (20.80)
West Europe/USA	530 (13.2)
East Europe	210 (5.2)
Experience (years)	
< 5 years	1050 (26.2)
5–10 years	1070 (26.8)
11–20 years	1050 (26.2)
> 20 years	830 (20.8)
Training status	
General practitioner	2730 (68.2)
Conservative dentistry	320 (8)
Endodontics	510 (12.8)
Prosthodontics	440 (11)
Working place	
Private clinic/center	3040 (76)
University	390 (9.8)
Ministry of health	370 (9.2)
Royal medical services	200 (5)

### Chief complain and patient risk assessment

As shown in (Table 3), Nearly half of the surveyed dentists focus on the chief complaint of their patients (50.8%) rather than doing full mouth assessment. Graduates from Asian and Arab countries other than Jordan and those working at Ministry of Health had the highest tendency to focus on chief complaint while graduates from West Europe and USA, prosthodontists, and those working at universities had the highest tendency to focus on full mouth assessment.

**Table 3:** Routine dental treatments and its association with sociodemographic variables

Condition	Treatment options	Total %	Gender		Country of last degree				Experience (years)				Training status				Working place			
			Male	Female	Jordan	Other Arab/Asian countries	West Europe/USA	East Europe	< 5	5–10	11–20	> 20	GDP	Conservative dentistry	Endodontics	Prosthodontics	Private clinic/center	University	MOH	RMS
		%																		
When treating a patient, I:	Focus on chief complaint	50.8	52.9	49.1	48.1	65.1	35.8	61.9	57.1	44.9	48.6	53	54.2	53.1	51	27.3	49.7	30.8	67.6	75
	Do full mouth assessment	49.2	47.1	50.9	51.9	34.9	64.2	38.1	42.9	55.1	51.4	47	45.8	46.9	49	72.7	50.3	69.2	32.4	25
	*P* value		0.454		**0.004**				0.312				**0.011**				**0.002**			
I rely in caries detection and diagnosis on:	Clinical	7.8	4.7	10.1	9.1	4.8	5.7	9.5	9.5	7.5	4.8	9.6	9.5	3.1	0	9.1	6.2	5.1	21.6	10
	Clinical & radiographs	63.5	63.4	63.6	65.4	60.2	62.3	57.1	72.4	68.2	71.4	36.1	61.2	71.9	62.7	72.7	63.2	66.7	54.1	80
	Clinical, radiographs, and other aiding diagnostic tools	28.8	32	26.3	25.5	34.9	32.1	33.3	18.1	24.3	23.8	54.2	29.3	25	37.3	18.2	30.6	28.2	24.3	10
	*P* value		0.089		0.594				**< 0.001**				0.109				**0.020**			
When treating incipient non-cavitated lesions, I use:	Preventive non-operative treatment	80.5	71.5	87.3	82.3	74.7	90.6	57.1	81.9	84.1	77.1	78.3	78.4	90.6	86.3	79.5	79.6	94.9	73	80
	Operative treatment	19.5	28.5	12.7	17.7	25.3	9.4	42.9	18.1	15.9	22.9	21.7	21.6	9.4	13.7	20.5	20.4	5.1	27	20
	*P* value		**< 0.001**		**0.005**				0.567				0.264				0.085			
When treating discolored demineralized occlusal fissures, I use:	Preventive non-operative treatment	33	36	30.7	35	27.7	39.6	14.3	38.1	29.9	40	21.7	30.8	25	41.2	43.2	32.9	43.6	24.3	30
	Class I cavity preparation	25.8	28	24.1	26.3	27.7	9.4	52.4	31.4	22.4	23.8	25.3	30.8	12.5	19.6	11.4	28	5.1	29.7	25
	Fissure sealant or PRR	41.2	36	45.2	38.7	44.6	50.9	33.3	30.5	47.7	36.2	53	38.5	62.5	39.2	45.5	39.1	51.3	45.9	45
	*P* value		0.185		**0.008**				**0.018**				**0.010**				0.088			
When treating lesions confined to enamel, I use:	Preventive non-operative treatment	34.8	30.2	38.2	34.2	22.9	56.6	33.3	33.3	35.5	31.4	39.8	28.9	50	49	43.2	33.2	53.8	32.4	25
	Operative treatment	65.2	69.8	61.8	65.8	77.1	43.4	66.7	66.7	64.5	68.6	60.2	71.1	50	51	56.8	66.8	46.2	67.6	75
	*P* value		0.099		**0.001**				0.671				**0.004**				0.057			
I routinely restore arrested asymptomatic lesions:	Yes	13.2	19.8	8.3	10.7	20.5	11.3	19	8.6	15	18.1	10.8	15.4	3.1	11.8	9.1	15.1	0	13.5	10
	No	28	22.7	32	27.6	27.7	39.6	4.8	27.6	27.1	28.6	28.9	24.9	28.1	29.4	45.5	27.3	33.3	29.7	25
	Only upon patient’s demand	58.8	57.6	59.6	61.7	51.8	49.1	76.2	63.8	57.9	53.3	60.2	59.7	68.8	58.8	45.4	57.6	66.7	56.8	65
	*P* value		**0.002**		**0.022**				0.507				0.068				0.290			
I treat radiographically detected proximal caries confined to enamel using:	Preventive non-operative treatment	44.8	36.6	50.9	47.3	27.7	64.2	33.3	50.5	40.2	45.7	42.2	39.6	59.4	52.9	56.8	46.7	53.8	21.6	40
	Operative treatment	55.2	63.4	49.1	52.7	72.3	35.8	66.7	49.5	59.8	54.3	57.8	60.4	40.6	47.1	43.2	53.3	46.2	78.4	60
	*P* value		**0.005**		**< 0.001**				0.465				**0.021**				**0.019**			
I treat deep dentinal caries with:	Removing carious soft and hard (infected and affected) dentin leaving caries-free stain-free floor	25.2	25	25.4	18.9	37.3	34.3	28.6	16.2	38.3	20	26.5	25.6	9.4	31.4	27.3	26.6	23.1	24.3	10
	Removing just the soft infected carious dentin leaving discolored hard affected dentin on the floor	74.8	75	74.6	81.1	62.7	66	71.4	83.8	61.7	80	73.5	74.4	90.6	68.6	72.7	73.4	76.9	75.7	90
	P value		0.920		**0.003**				**0.001**				0.145				0.409			
My routine treatment of old failed restorations:	Replace	67.2	70.3	64.9	68.3	75.9	54.7	52.4	72.4	77.6	64.8	50.6	67.8	56.2	80.4	56.8	68.8	61.5	54.1	80
	Repair if possible	32.8	29.7	35.1	31.7	24.1	45.3	47.6	27.6	22.4	35.2	49.4	32.2	43.8	19.6	43.2	31.2	38.5	45.9	20
	*P* value		0.251		**0.032**				**0.001**				**0.047**				0.152			
My routine treatment of old stained restorations:	Keep and polish	81.5	78.5	83.8	81.9	78.3	84.9	81	81.9	82.2	77.1	85.5	81.7	87.5	70.6	88.6	81.6	89.7	81.1	65
	Replace	18.5	21.5	16.2	18.1	21.7	15.1	19	18.1	17.8	22.9	14.5	18.3	12.5	29.4	11.4	18.4	10.3	18.9	35
	*P* value		0.178		0.802				0.518				0.099				0.146			
My treatment of old restorations without clinical or radiographic signs of failure:	I convince the patient to keep them	84.8	83.1	16.9	86.4	78.3	86.8	85.7	84.8	83.2	85.7	85.5	83.2	96.9	88.2	81.8	83.9	84.6	91.9	85
	I replace them upon patient’s demand	15.2	86	14	13.6	21.7	13.2	14.3	15.2	16.8	14.3	14.5	16.8	3.1	11.8	18.2	16.1	15.4	8.1	15
	*P* value		0.436		0.338				0.956				0.175				0.651			

*P* values of Chi-square test

### Caries diagnostic tools

The majority (63.5%) of dentists rely on both clinical examination and radiographs in caries detection and diagnosis. Minority rely only on clinical examination while around one third may seek other aiding diagnostic tools.

### Incipient lesions treatment

When treating incipient non-cavitated lesions, the majority (80.5%) use preventive non-operative treatment rather than operative treatment (19.5%). Preventive treatment was used more frequently by female dentists and graduates from West Europe and USA and least frequently by graduates from East Europe.

### Discolored fissures treatment

When treating discolored occlusal fissures, most dentists use fissure sealants or preventive resin restoration (41.2%), while the rest use preventive non-operative treatment (33%) or Class I cavity preparation (25.8%). Class I cavity preparation was used more frequently by graduates from East Europe and least frequently by graduates from West Europe and USA and those specialized in conservative dentistry and prosthodontics. When experience was taken into account, those who had experience more than 20 years showed the highest tendency to use fissure sealants or preventive resin restoration compared to those with less than 20 years of experience.

### Enamel lesions treatment

Nearly two-thirds of dentists (65.2%) treat lesions confined to enamel with operative treatment. Graduates from West Europe and USA and specialists in conservative dentistry and endodontics utilize preventive treatments more frequently.

### Arrested lesions

Of the surveyed dentists, 13.2% routinely restore, 28% do not routinely restore, and 58.8% only restore upon patient’s demand arrested asymptomatic carious lesions. Higher frequency of males and lower frequency of graduates from West Europe and USA routinely restore these lesions.

### Proximal caries treatment

When asked about the routine management of radiographically detected proximal caries confined to enamel, 44.8% use preventive treatment while higher frequency (55.2%) use operative treatment. Preventive treatment was the choice favored by females, graduates from West Europe and USA, dentists with a specialty, and those working at universities.

### Deep dentinal lesions treatment

Nearly three-quarters of dentists (74.8%) treat deep dentinal caries by removing just the soft carious dentin while 25.2% remove additionally the discolored hard dentin on the floor. Removing the hard dentine was practiced more frequently by graduates from Arab countries other than Jordan and Asian countries and less frequently by fresh graduates.

### Repair versus replacement

The routine treatment of old failed restorations was replacement by two-thirds of dentists (67.2%) or to repair if possible (32.8%). Graduates from East and West Europe and USA, those with more than 20 years of experience, those specialized in conservative dentistry and prosthodontics tend more frequently to repair these restorations. The routine treatment of old stained restorations was to keep and polish by 81.5% of dentists or replacement (18.5%), without significant effects of the sociodemographic variables.

Of the survey dentists and without significant effects of the sociodemographic variables, 84.8% convince the patients to keep old restorations without clinical or radiographic signs of failure, while 15.2% replace them upon patient’s demand.

## Discussion

In the current study the null hypothesis was accepted. There was no correlation between EBD in caries research and decision making in clinical practice in operative dentistry for dentists in Jordan. The good news is the partial presence of these research-based cariology concepts among our dentists.

Treatment decision was deceptively simple when dental caries was equated to just a cavity in the tooth and treatment was equated to just filling the cavity [17]. Unfortunately, this mechanical solution for a biological problem has prevailed for centuries [18]. However, with the current changeover in all dimensions of dental caries, caries management became dichotomous, in which treatment decision shifts from the surgical model towards the medical model and bifurcates to identify and eliminate causes, and to manage and treat signs and symptoms [17–19]. Therefore, there is a growing consensus that the current surgical model focusing on drilling and filling the tooth confined to operative treatment, should be replaced with individualized comprehensive patient treatment plan encompasses prevention as non-operative treatment [20, 21].

Therefore, in the present era, productive and desirable changes are mandatory in the daily practice clinical decisions in which the practice of dentistry emphasizes or should emphasize more on preventive non-operative treatment and remineralization of demineralized tooth structure rather than simply drilling and filling teeth [11, 22].

In 1995 Pitts and Longbottom have proposed a blue-print for an approach to categorize caries by the management option appropriate for carious lesions to facilitate standard communication regarding clinical decision in caries management whether preventive care (PCA) or operative care (OCA) to be advised (PCA and OCA approach) [23]. However, the adoption of any diagnostic and management system can’t be done independent from other individual-environmental caries-associated risk factors [12]. Unfortunately, nearly half of the surveyed dentists focus on the chief complaint of their patients rather than doing full mouth assessment. Although the well-know and widespread ICDAS guidelines emphasized the importance of caries risk factors assessment when constructing personalized dental treatment plan [7]. Undergraduate curriculum, postgraduate studies, and healthcare system play pivotal role in adopting such philosophy.

Preventive strategies when treating incipient non-cavitated lesions and discolored occlusal fissures also depend on the dentists’ educational undergraduate and postgraduate background. The majority of our dentists who were graduated from West Europe and USA and those who are specialists use preventive non-operative treatment rather than operative treatment in treating incipient non-cavitated lesions, discolored demineralized occlusal fissures, lesions confined to enamel, and arrested asymptomatic carious lesions. Nevertheless, nearly two-thirds of our dentists treat lesions confined to enamel with operative treatment, restore arrested asymptomatic lesions, and replace old restorations rather than repairing them, and nearly half of them treat radiographically detected proximal caries confined to enamel operatively. Thus, despite the introduction of the Caries Management Pathways and Caries Management Cycle, to facilitate accomplishment and implementation of MID, tooth structure preservation, and prevention strategies in practice universally [11, 12], those preventive strategies have not been widely adopted because the cultural background of dental education for both dentists and patients are focused on operative restorative procedures rather than non-operative preventive ones [11].

The most common caries detection method is the combination of visual-tactile examination with bitewing radiography. Bitewing radiographs were used based on the argument that they are more sensitive than clinical inspection for detecting approximal lesions and for occlusal lesions in dentin, for estimating depth of the lesion, and for monitoring lesion behavior [24]. The majority of our dentists rely on clinical examination and radiographs in caries detection and diagnosis. Half of them chose to restore radiographically detected proximal caries confined to enamel.

The assessment of lesion activity is also very important when using ICDAS to help on the treatment decisions, particularly when preventive options should be implemented. Thus, regardless which caries detecting method is used by the clinician it is of pivotal importance to implement it in detecting early remineralizable non-cavitated lesions to help adopting preventive non-operative strategy rather than pointing out lesions to be operatively restored (drill and fill strategy).

One of the key concepts of MID is to prevent extension and preserve tooth structure [6], which created a pathway for preventive resin restoration and fissure sealants in treating discolored demineralized occlusal fissures. Only one-quarter of the surveyed dentists operated demineralized occlusal fissures as class I cavity preparation while less than half of them used fissure sealants or preventive resin restoration, and approximately one-third used preventive non-operative treatment. These findings are not surprising since several studies have demonstrated that regardless whether dentists accept or reject evidence-based treatment recommendations, they barely adopt them in decision making in their clinical practice [25–28].

The same scenario applies when treating arrested asymptomatic carious lesions. Still some dentists routinely restore these lesions, and it is common to restore them upon patient’s demand. Histologically, an arrested dentinal lesion is localized between two highly mineralized layers; the hypermineralized surface layer with heavily mineralized intertubular dentin and the sclerosed white opaque sclerotic zone filled with calcified contents [29]. The dark brown discoloration in such lesions is thought to be due to the melanin from amino acids and carbohydrates derivatives, and degenerated bacteria or/and their proteins and nucleic acids degradation products [30]. The Millard reaction (sugar-protein reaction) which is suggested to be responsible for lesion discoloration, modifies amino acids in collagen making them more resistant to enzymatic degradation and proteolytic attacks [31].

When treating deep dentinal carious lesions three-quarter of the surveyed dentists removed just soft infected carious dentin while one-quarter removed the discolored hard affected dentin, too. The good news is not only adopting a more conservative approach toward treating deep dentinal carious lesions, but also that fresh graduates practiced it more and halted affected demineralized dentin which is amenable to remineralization.

One of the basic principles of MID is repairing rather than replacing old restorations. This approach will not only prevent sacrificing healthy dental tissue, it will also delay the restoration death spiral [32, 33]. Replacing a restoration was defined as the entire removal of the existing defective/failed restoration and any adjacent pathologically altered and discolored tooth tissue that was esthetically or functionally unacceptable [32], while repairing it means the partial replacement of a restoration that presents no clinical or radiographic evidence of failure [34]. A better understanding of dentist, patient, and restoration related variables that are associated with the decision to repair or replace defective restorations may assist with the development of guidelines to improve treatment of existing restorations [32, 35].

While two-thirds of our dentists routinely replace old failed restorations, more than three-quarter of them keep and polish old stained restorations and convince their patients to keep old acceptable restorations. Repairing old restorations whether failed or not rather than replacing them was proved to be an effective non-invasive treatment choice in modern dentistry [35, 36]. Although repairing old restorations has become an integral part of MID and it is gaining increased acceptance among dental practitioners and patients, repair of restorations is not practiced frequently worldwide due to several factors such as; lack of global guidelines, limited information on the long-term clinical outcomes, continuous changes in materials and technologies, and variations in dental teaching curricula worldwide as well as healthcare systems [37, 38].

The null hypothesis in the current study was accepted in most of its facets, indicating a gap between evidence-based research and clinical practice in terms of cariology and operative dentistry. On the other hand, in our previous study investigating various restorative options (esthetic treatment, replacement of missing teeth, endodontic treatment, and treatment of badly damaged teeth), the null hypothesis was rejected in most of its facets [16]. This variation in treatment strategies among the same population maybe attributed to different dental schools and curriculums, personal experience and knowledge, or it may be due to absence of definitive universal evidence-based clinical guidelines.

To make it easier, international, and less confusing to dentists the CariesCare practice guide was derived from ICCMS in 2019. It is based on collecting and summarizing best practice as informed by the best available evidence. This practice-friendly consensus guide enables clinicians all over the world to implement evidence-based relevant information into their daily dental practice. It promotes a patient-centered, risk-based approach to caries management, leading to personalized intervention through a four-step process (4D cycle) [39].

The emergence of COVID-19 pandemic has disclosed a further essential need for MID and empathized the importance of incorporating the Minimal Invasive Oral Care (MIOC) term in primary dental care in daily clinical practice and across all dental disciplines [40]. We now appear to be at a unique point in time when many interests and opportunities align and global changes in health policy at WHO after the pandemic advocating strategies for improving oral health and seeking to reorient the traditional curative approach towards a preventive approach contributing to the improvement of the oral health of the population with a positive impact on overall health [41].

The limitations of the current study in aspect of gathering data due to Covid-19 pandemic, in which collecting data solely depended on electronic survey. The questionnaire was designed to be as concise as practical to insure a high rate of responses. More sophisticated investigations that can lead to a deeper insight to the study can be carried on later. In term of statistical analysis, descriptive statistics were used as this research was the first time to be done in cariology era in Jordan.

## Conclusion

In operative dentistry, the evidence-based research is not implemented clinically. On the whole, nearly half of our dentists adopt an operative restorative strategy rather than a preventive non-operative philosophy when treating patients’ carious lesions, focusing on treating the sign on a tooth rather than exploring the cause for the patient.

### Recommendation

To optimize relationship between evidence-based dentistry and clinical decision-making, more emphasis has to be placed on communicating research data to educators to integrate them in dental curriculum. Incorporating the European Core Curriculum in Cariology for undergraduate dental students, which was developed by the Association for Dental Education in Europe (ADEE) together with the European Organization for Caries Research (ORCA) in our dental schools is a fundamental step in the right direction [42].

## Data Availability

The datasets used and/or analyzed during the current study are available from the corresponding author on reasonable request.

## Abbreviations

**MID**: Minimal invasive dentistry
**EBD**: Evidence-based dentistry
**JDA**: Jordan Dental Association
**PCA**: Preventive care advised
**OCA**: Operative care advised
**ICDAS**: International Caries Detection and Assessment System
**ICCMS**: International Caries Classification and Management System
**MIOC**: Minimal Invasive Oral Care
**ADEE**: Association for Dental Education in Europe
**ORCA**: European Organization for Caries Research
